# Anxiolytic Effects of 8-O-Acetyl Shanzhiside Methylester on Acute and Chronic Anxiety via Inflammatory Response Inhibition and Excitatory/Inhibitory Transmission Imbalance

**DOI:** 10.1007/s12640-020-00203-2

**Published:** 2020-05-04

**Authors:** Ting Sun, Li Luo, Qin-Qin Tian, Wen-Ju Wang, Qing-Qing Liu, Le Yang, Kun Zhang, Wei Zhang, Ming-Gao Zhao, Qi Yang

**Affiliations:** 1 Precision Pharmacy & Drug Development Center, Department of Pharmacy, Tangdu Hospital, the Fourth Military Medical University, Xi’an, 710038 Shaanxi Province People’s Republic of China; 2 Department of Chemistry, School of Pharmacy, the Fourth Military Medical University, Xi’an, 710032 China; 3 Student Brigade, the Fourth Military Medical University, Xi’an, 710032 China; 4 Department of Pharmacy, Xijing Hospital, the Fourth Military Medical University, Xi’an, 710032 China

**Keywords:** Anxiety, 8-O-Acetyl shanzhiside methylester, Excitatory/inhibitory balance, Basolateral amygdala, Inflammatory response

## Abstract

Anxiety leads to a global decline in quality of life and increase in social burden. However, treatments are limited, because the molecular mechanisms underlying complex emotional disorders are poorly understood. We explored the anxiolytic effects of 8-O-acetyl shanzhiside methylester (8-OaS), an active component in *Lamiophlomis rotata* (*L*. *rotata*; Benth.) or Kudo, a traditional herb that has been shown to be effective in the clinical treatment of chronic pain syndromes in China. Two mouse anxiety models were used: forced swimming stress (FSS)–induced anxiety and complete Freund’s adjuvant (CFA)–induced chronic inflammatory pain. All animal behaviors were analyzed on the elevated plus maze and in the open-field test. 8-OaS significantly ameliorated anxiety-like behaviors in both anxiety models and inhibited the translation enhancement of GluN2A, GluN2B, and PSD95. Moreover, a reduction in GABA receptors disrupted the excitatory/inhibitory (E/I) balance in the basolateral amygdala (BLA), indicated by increased excitatory and decreased inhibitory presynaptic release. 8-OaS also blocked microglia activation and reduced the phosphorylation of p38, c-Jun N-terminal kinase (JNK), NF-κB p65, and tumor necrosis factor alpha (TNF-α) in the BLA of anxiety mice. 8-OaS exhibits obvious anxiolytic effects by regulating the excitatory/inhibitory (E/I) synaptic transmission and attenuating inflammatory responses in the BLA.

## Introduction

Anxiety disorder is highly common worldwide, with an estimated prevalence of 15% in developed countries, and seriously affects people’s life and work (Wu et al. 2008). Anxiety disorder is a chronic and functional disability with high psychological pressure, characterized by overwhelming stress, attention difficulties, and physiological symptoms such as muscle tension and insomnia (Beery and Kaufer 2015; Du et al. 2019). Antidepressants and benzodiazepines are clinically useful for the treatment of anxiety, but can lead to considerable side effects such as liability of physical dependence, addiction, excessive sedation, and abuse (Locke et al. 2015). Better anxiolytic drugs with fewer side effects are therefore needed.

In clinical experiments, acute stress, chronic inflammation, and pain cause anxiety behaviors. Stress is an established and pivotal precipitating factor for several neuropsychiatric diseases, especially for anxiety and mood disorders (Thakare et al. 2017). Animal studies have shown that stress can induce various alterations of neurotransmission systems, and hyperexcitation can promote anxiety-like behavior in the basolateral amygdala (BLA), because of enhanced excitatory glutamate or reduced inhibitory GABA transmission (Liu et al. 2012; Stanika et al. 2009). The imbalance between excitation and inhibition in neural circuits is therefore highly relevant to the occurrence and development of anxiety. Additionally, neuroimaging studies show that an increase in inflammation is associated with enhanced threat- and anxiety-related brain circuitry, particularly activation of the amygdala (Tasan et al. 2010). Moreover, animal studies have demonstrated an increase in anxiety-like behaviors during experimentally induced systemic inflammatory responses (Gallagher et al. 2019). Regulating the excitatory and inhibitory transmission balance as well as inflammation may thus be an effective method for treating anxiety disorders.

*Lamiophlomis rotata* (*L*. *rotata*) Kudo (“Duyiwei” in Chinese) is a Chinese folk medicinal plant from Xi-zang (Tibet), which is traditionally used to relieve pain, detumescence, and hemostasis; reinforce marrow; and promote blood circulation to remove blood stasis (Jiang et al. 2010; Yi et al. 1997). The active ingredients of *L*. *rotata* are iridoid glycosides, mainly shanzhiside methylester (SM) and 8-O-acetyl-SM (8-OaS) (La et al. 2015; Shang et al. 2011). Emerging studies suggest that 8-OaS has neuroprotective effects on hypoxia and glucose deficiency by suppressing inflammatory and apoptosis-related cascade reactions (Jiang et al. 2010; Jiang et al. 2011). In addition, the main mechanism of 8-OaS analgesia is the regulation of nociceptive information transmission (related to the deactivation of the NMDAR/PKC and NO/cGMP/PKG pathways) and spinal neuroinflammatory responses (decrease in TNF-α and IL-1 β, increase in IL-10 production) (Zheng et al. 2011). 8-OaS inhibits the production of TNF-α and other pro-inflammatory cytokines and reduces the phosphorylation of P38 MAPK and NF-κB in neuropathic pain models (Fan et al. 2016; Ji et al. 2014; Xu et al. 2006; Zhu et al. 2014). These effects of 8-OaS are correlated with the inhibition of the inflammatory response. However, its anxiolytic effects in a model of acute stress and inflammation as well as the involved mechanisms remain unclear.

This study thus aimed to investigate the roles and mechanism of 8-OaS in the modulation of anxiety-like behaviors, in two animal models of anxiety, an acute forced swimming stress (FSS) model and a chronic CFA-induced inflammatory pain model.

We found that 8-OaS exerted anxiolytic-like effects in the open-field test (OFT) and the elevated plus maze (EPM) anxiety tests in both models. The correlative proteins involved in the balance between excitation and inhibition, including GluN2A, GluN2B, PSD95, and subunits of GABAA receptors (GABAAα2 and GABAAγ2 in FSS-induced mice, and inflammatory signaling proteins including phospho-JNK, phospho-P38, IL-6, TNF-α, and NF-κB p65) were sequentially examined following 8-OaS administration in CFA-injected mice. Our results clarify the role of 8-OaS in stress- and CFA-induced mood disorders and suggest 8-OaS as a potential therapeutic target for the treatment of clinical anxiety.

## Materials and Methods

### Animals

Six- and 8-week-old C57BL/6 male mice were provided by the Laboratory Animal Center of Fourth Military Medical University (FMMU). The animals were housed in a colony room at 24 ± 2 °C and 50–60% humidity and under a 12-h light/dark cycle (light on at 7:00 AM) with food and water available ad libitum. All behavioral tests were conducted during the light period. Mice were adapted to the laboratory conditions for at least 1 week before testing. All procedures were approved by the FMMU Animal Care Committee.

### Chronic Inflammatory Pain Model (Model 1)

As in a previous study (Wang et al. 2015), the left hind paw plantar of test mice was injected with CFA (10 μl, 50% in saline) to induce persistent inflammatory pain. The same volume of 0.9% saline was injected into the left hind paw plantar of control mice.

### Forced Swimming Test (Model 2)

The forced swimming test was conducted in an open cylindrical container (diameter 10 cm, height 25 cm) filled with water at 23–25 °C for 15 min. Mice were placed in separate containers and forced to swim without their tails touching the bottom. The animals were forced to swim for 15 min a day for 2 consecutive days. At the end of each session, the mice were taken out of the water and dried immediately.

### Experimental Design and Drug Treatment

8-OaS (purity > 98%) was purchased from Shanghai Pure One Biotechnology (Shanghai, China), dissolved in 0.9% saline containing 10% dimethylsulfoxide (DMSO), and administered intraperitoneally (*i.p.*) at different doses (0.02, 0.2, 2 mg/kg) after the CFA insult. The 8-OaS doses used here were based on earlier research (Zhang et al. 2018). Mice in the control group were treated with saline for 2 weeks (from day 1 to day 14). Mice in the chronic inflammatory pain model groups (*n* = 6 per group) were continuously injected with 8-OaS or saline for 2 weeks (from day 1 to day 14), and all behavior tests were conducted on day 15 after CFA injection. In the acute stress model groups, mice were continuously injected with 8-OaS or saline for 3 days (from day 1 to day 3), and behavior tests were conducted on day 4. After the behavior tests, all animals were sacrificed, and the BLA was collected to detect the molecular mechanism underlying the effects of 8-OaS administration during phases of anxiety.

### Open-field Test

The open-field test (OFT) was performed as depicted previously (Yang et al. 2016). An individual animal was placed in the center area of the box for 15 min. Exploratory behaviors were recorded using a camera fixed above the floor and analyzed with a video-tracking system. The OFT test was performed before the EPM test but on the same day.

### Elevated Plus Maze Test

To assess anxiety-like behaviors, EPM tests were also performed as detailed in a previous report (Sun et al. 2016). The apparatus contained two opposing open arms (25 × 8 × 0.5 cm^3^) and two closed arms (25 × 8 × 12 cm^3^) extending from a common central zone (8 × 8 cm^2^). The EPM was located 50 cm above the floor. For each test, an individual animal was initially placed in the central area facing an open arm and allowed to move freely for 5 min while being recorded with a camera fixed above the maze.

### Immunohistochemistry Staining

After the behavior tests, the brain slices for immunohistochemistry staining were prepared from the intracardially perfused brains, and staining procedures were used as described previously (Sun et al. 2018). All frozen brain sections were washed with 0.3% Triton X-100 PBS and then blocked (10% goat serum, 0.1% Triton X-100 in PBS) for 2 h at 4 °C. Slices were incubated with mouse goat anti-Iba-1 (1:1000) in blocking solution for 24 h at 4 °C. Next, the sections were rinsed with PBS and incubated with donkey anti-goat IgG Alexa Fluor 594 (1:200) in PBS for 1 h at room temperature. All antibodies were diluted in PBS with 0.1% Triton X-100 and 2% bovine serum albumin. Nuclei were counter-stained with Hoechst33258 (1:100). The brain slices were then moved to slides, coverslipped with 50% glycerin, and photographed with a FluoView FV100 microscope (Olympus).

### Enzyme-Linked Immunosorbent Assay

After the behavior tests, the BLA tissue samples were removed from the brains of the dissected mice. According to the manufacturer’s instructions (R&D Systems, Minneapolis, MN), levels of the inflammatory cytokines interleukin 1β (IL-1β), interleukin 6 (IL-6), and TNF-α were detected in the BLA using a double-antibody sandwich method.

### Determination of GABA and Glutamate

After treatment with 8-OaS for 3 days, GABA and glutamate were detected using high-performance liquid chromatography (HPLC; Agilent Technologies 1260 Infinity, Agilent Technologies, Wilmington, DE), according to previously described methods (Wang et al. 2017). Before derivatization, the samples were dissolved with boracic acid buffer (pH 9.0) and centrifuged for 15 min at 3000 r/min at 4 °C. Samples were mixed with 2,4-dinitrofluorobenzene (DNFB) and 0.5 mol/L NaHPO_3_ buffer for 1 h at 60 °C; then, a phosphate buffer solution (pH 7) was added to stop the reaction. Samples were analyzed using a UV detector (360 nm, Agilent Technologies 1260 Infinity). The mobile phase was KH_2_PO_4_ buffer (pH 6.0), acetonitrile, and ddH_2_O (84:8:8, v/v/v) at a flow rate of 1.0 mL/min. A Themo TC-C18 column (250 × 4.6 mm^2^; particle size 5 mm) was used. The concentrations were obtained using the LC solution software (Shimadzu) based on standard substances.

### Western Blot Analysis

Samples from BLA tissues were harvested after various treatments according to our previously reported method (Yang et al. 2016). The western blot analysis determined the expression levels of GluA1 (1:1000; Abcam, ab31232), phosphorylated forms of GluA1 including phospho-GluA1-831 (1:1000; Abcam, ab5847) and phospho-GluA1-845 (1:1000; Abcam, ab5849), GluN2A (1:1000; Abcam, ab133265), GluN2B (1:1000; Millipore, Billerica, MA; MAB5780), PSD95 (1:1000; Abcam; ab2723), phosphorylated forms of JNK (1:1000; Cell Signaling Technology; Danvers, MA) and P38 (1:1000; Cell Signaling Technology), TNF-α (1:1000; Cell Signaling Technology), and p65 (1:1000; Cell Signaling Technology); β-actin (1:10,000; Sigma, St. Louis, MO) served as a loading control. The target protein signal was detected and digitized using ECL solution and ImageJ software (NIH, Bethesda, MD).

### Statistical Analysis

Data were analyzed with SPSS 19.0 and expressed as means ± SEM. Comparisons between the two groups were conducted using independent sample *t* tests. Results of multiple groups were analyzed using one-way ANOVA followed by least significant difference (LSD) tests. In all cases, statistical significance was accepted at *p* < 0.05.

## Results

### 8-OaS Relieves Anxiety-Like Behaviors Induced by Forced Swimming Stress

**Fig. 1:**
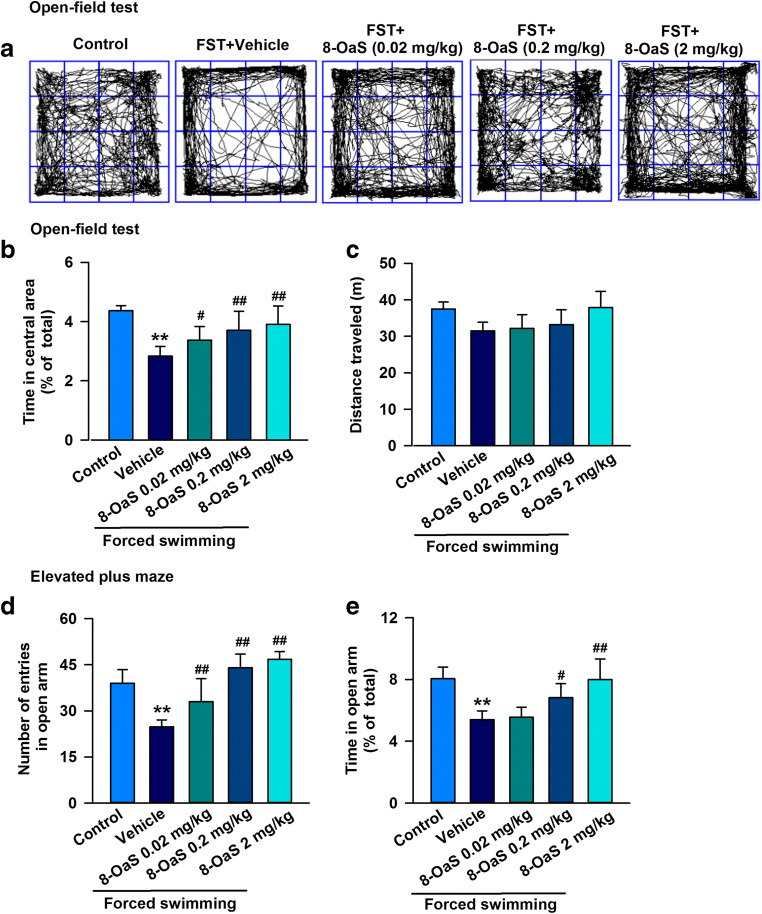
8-OaS relieved anxiety-like behaviors in FSS-induced mice. Representative traces in the OFT during a period of 15 min. Behavioral tests were performed on day 14. **a**, **c** In the OFT, 8-OaS administration (0.02, 0.2, 2 mg/kg) for 14 days significantly increased **b** the time spent in the central area but had no effect on **c** the total distance traveled. In the EPM test, 8-OaS treatment reversed **d** the number of entries into and **e** time spent in the open and closed arms. Each value represents the mean ± SEM of three independent experiments (*n* = 12, *p < 0.05, **p < 0.01 versus the control group; ^#^*p* < 0.05, ^##^*p* < 0.01 versus the FSS group)

The FSS model was used to investigate the importance of 8-OaS in acute stress–induced anxiety-like behaviors. Anxiety-like behaviors were assessed with the EPM and OFT, which are widely used for this purpose in rodents. In the OFT, compared with the control group, the stress group displayed an obvious decrease in the time spent in the central area, while the total distance traveled showed no change (Fig. 1a, b). In the EPM test, the stressed group showed a remarkable decrease in the number of entries and the time spent in the open arms (Fig. 1a, b), although the total distance traveled showed no significant change compared with the vehicle group (Fig. 1c). Administration of 8-OaS for 3 days remarkably improved anxiety-like behaviors in these stressed mice, as our results show that the number of entries into and the time spent in the open arms in the EPM test increased (Fig. 1d, e) and that the mice spent more time in the central square in the OFT (Fig. 1b). These results suggest that 8-OaS is involved in anxiolytic responses that reverse anxiety-like behaviors after acute stress.

### 8-OaS Attenuates CFA-Induced Anxiety-Like Behaviors in the BLA

**Fig. 2:**
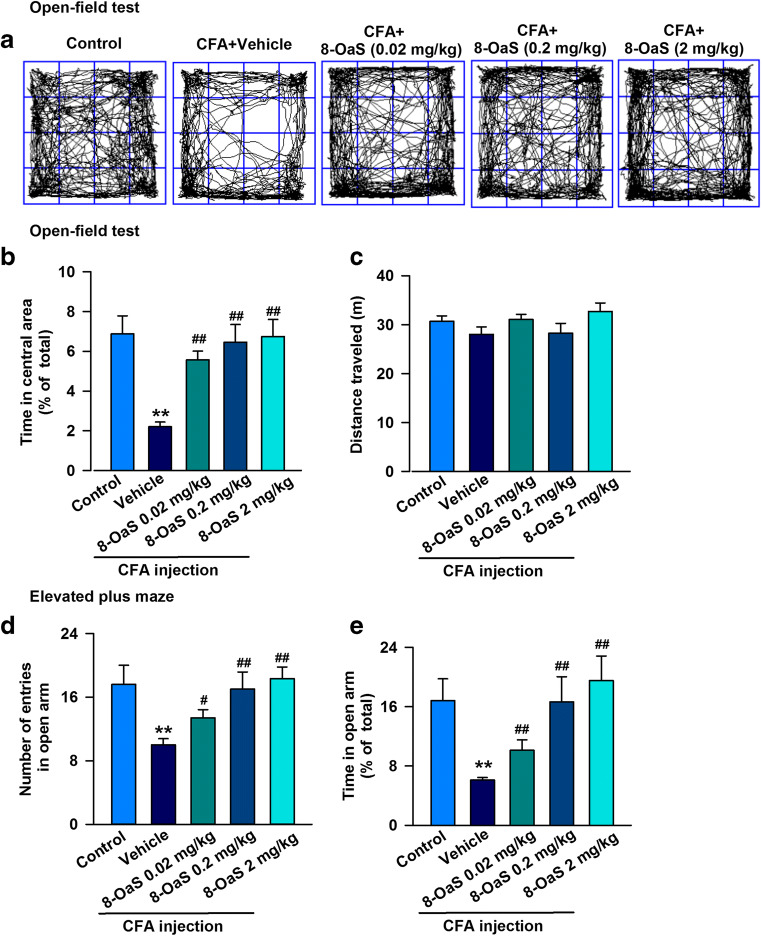
8-OaS attenuated anxiety-like behaviors in CFA-injected mice. **a** Representative traces in the OFT during a period of 15 min. Behavioral tests were performed on day 3. **b** In the OFT, 8-OaS treatment (0.02, 0.2, 2 mg/kg) for 3 days remarkably reversed the time in the center area in CFA-injected mice; however, no difference in **c** the total distance traveled was observed in either group in the OFT. **d** In the EPM test, 8-OaS (0.02, 0.2, 2 mg/kg) increased the number of entries into and time spent in the open arms in CFA-injected mice. Each value represents the mean ± SEM of three independent experiments (*n* = 12, *p < 0.05, **p < 0.01 versus the control group; ^#^*p* < 0.05, ^##^*p* < 0.01 versus the CFA-injected group)

CFA was injected to induce chronic inflammation–mediated anxiety in mice. Different behaviors were assessed using the OFT, and we found that the time spent in the central zone was reduced in CFA-injected mice compared with that in the vehicle group (Fig. 2a, b). However, there were no obvious changes in the total distance traveled (Fig. 2c). In the EPM, our data shows that CFA induced anxiety-like behaviors and that mice made less entries into the open arms (Fig. 2d) and spent less time in the open arms (Fig. 2e) at 14 days after CFA injection, compared with the control group. Treatment with 8-OaS for 2 weeks significantly ameliorated the CFA-induced anxiety-like behaviors in mice and increased the number of entries and the time spent in the open arms in the EPM test (Fig. 2d, e) as well as the time spent in the central square in the OFT (Fig. 2b). These observations further confirm that 8-OaS treatment leads to anxiolytic effects on CFA-induced inflammatory pain.

### 8-OaS Maintains GABAergic and Glutamatergic Transmission Balance

The excitatory/inhibitory (E/I) balance of neural activities is necessary for all central physiological functions. An imbalance in E/I signaling leads to seizures, schizophrenia, autism, and anxiety (Lucchetti et al. 2013). To investigate the levels of neurotransmitters in the BLA, we specifically checked glutamate and GABA concentrations using HPLC. Chromatograms of glutamate and GABA yielded peaks at 11.28 min and 22.27 min, respectively (Fig. 3a). The FSS group showed higher glutamate (8.21 ± 0.60 nmol/mg) and lower GABA (1.31 ± 0.08 nmol/mg) content in the BLA than in the vehicle group (glutamate 5.91 ± 0.52 nmol/mg; GABA 1.75 ± 0.09 nmol/mg) (Fig. 3b, c). Glutamate levels were lower after 8-OaS treatment (Fig. 3b). In contrast, GABA levels were enhanced after 8-OaS administration compared with those seen in control group mice (Fig. 3c). These data indicate that treatment with 8-OaS reversed the abnormal release of neurotransmitters in the BLA that had been induced by acute stress.

**Figure:**
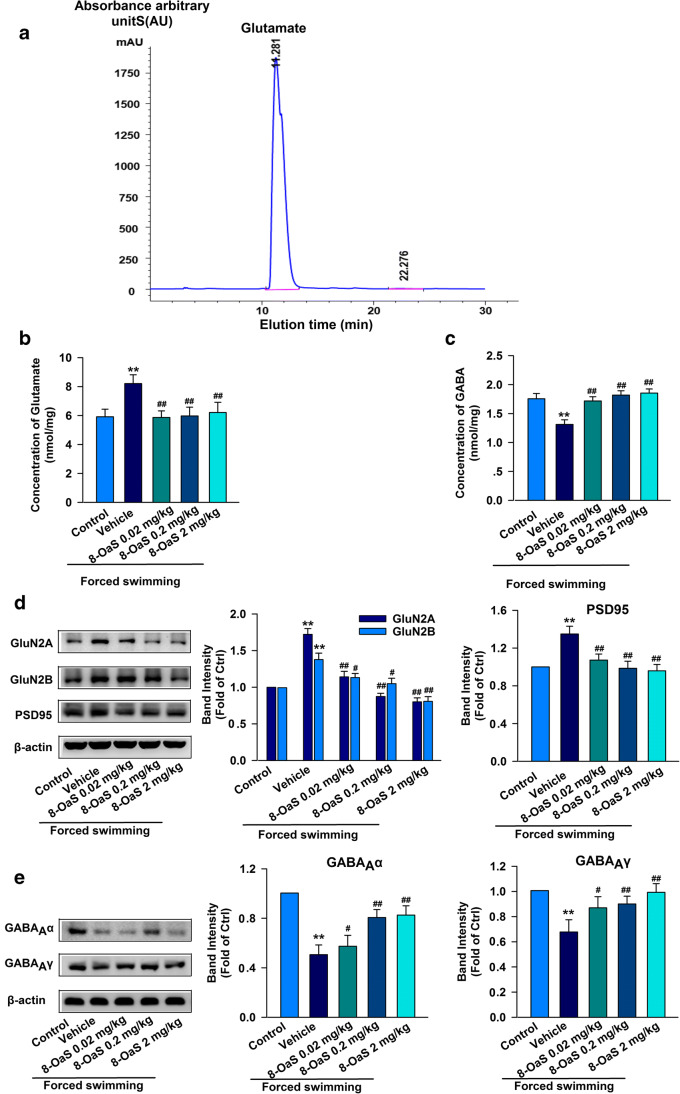

Postsynaptic receptors also play a vital role in the excitation/inhibition of signal transduction between neurons. Excitatory postsynaptic receptors include NMDA and AMPA receptors. Excitatory postsynaptic receptors are mainly GABA_A_ receptors. Thus, we determined the expression changes in receptor subunits in the BLA after acute stress. The levels of GluN2A, GluN2B, and PSD95 (Fig. 3d) were remarkably increased in the BLA. Administration of 8-OaS for 3 days obviously decreased the elevation of GluN2A, GluN2B, and PSD95 (Fig. 3d) in the BLA of acutely stressed mice. Next, two lowly expressed subunits of GABAA receptors—GABAAα2 and GABAAγ2—were examined in the FSS group, compared with that in the vehicle group (Fig. 3e), and were found to be enhanced after 8-OaS administration for 3 days (Fig. 3e). These results suggest that 8-OaS treatment ameliorated the imbalance between GABAergic and glutamatergic transmission in the BLA of FSS mice.

### Anti-inflammatory Effects of 8-OaS in the Mouse BLA

**Fig. 4:**
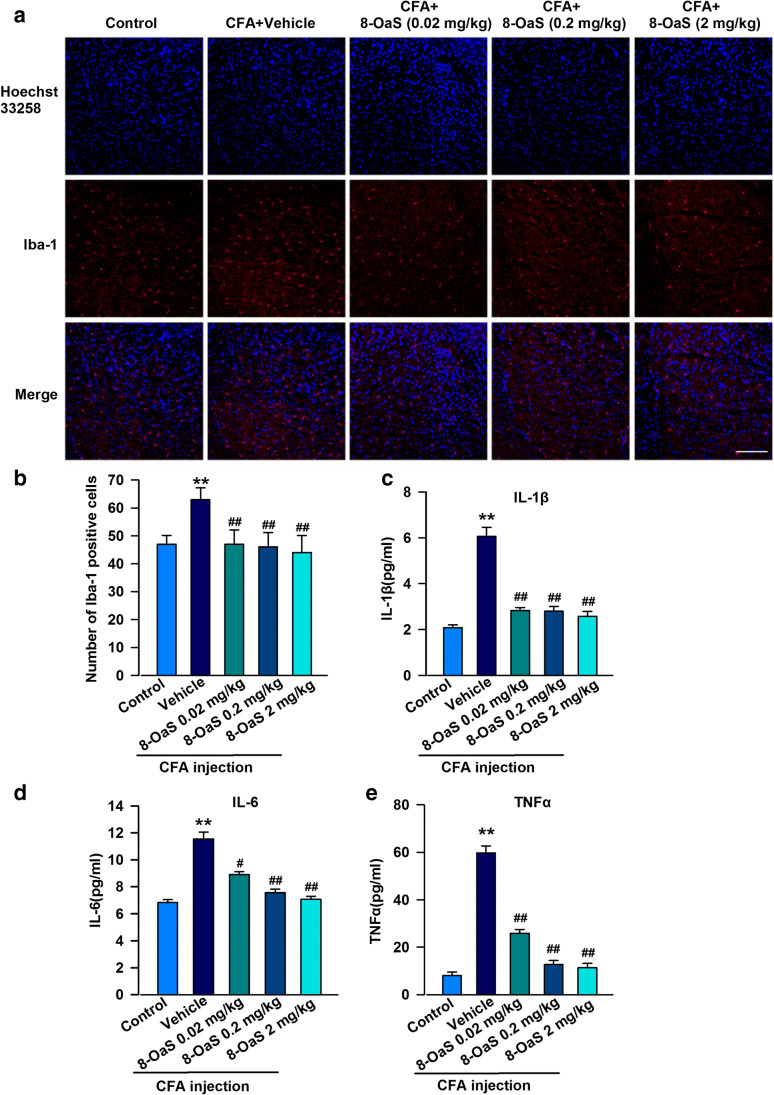
The effects of 8-OaS on inflammation in the mouse BLA during CFA-induced chronic inflammatory pain. **a** After 14 days, the brain slices containing BLA were immunostained with microglial marker Iba-1 antibody (red), and nuclei were stained with Hoechst33258 (blue). Scale bar = 20 μm. **b** 8-OaS inhibited the activation of microglia in the BLA of CFA-injected mice and had a dose-dependent effect. 8-OaS decreased the elevated levels of **c** IL-1β, **d** IL-6, and **e** TNF-a in the BLA on day 14 after CFA injection. Each value represents the mean ± SEM of three independent experiments (*n* = 6, *p < 0.05, **p < 0.01 versus the control group; ^#^*p* < 0.05, ^##^*p* < 0.01 versus the CFA-injected group)

Microglia are responsible for immune responses upon inflammatory stimulation via the production of cytokines and chemokines. The chronic inflammation caused by CFA injection can activate the microglia in the BLA area. The morphological features of the activated microglia are enlargement and roundness, and the expression ratio of the specific marker Iba-1 increases (Fig. 4a, b), which can then drive the secretion of cytokines that mediate inflammatory responses in the brain. The fluorescence intensity of Iba-1 was markedly decreased after treatment with 8-OaS for 14 days. This finding suggests that 8-OaS reduced the activation of immune cells in the brain after CFA challenge.

To further identify the roles of 8-OaS on inflammation, the concentrations of inflammatory cytokines in the BLA were analyzed by an enzyme-linked immunosorbent assay. Concentrations of pro-inflammatory cytokines, such as IL-1β (6.065 ± 0.353 pg/mL), IL-6 (11.55 ± 0.504 pg/mL), and TNF-α (59.722 ± 2.945 pg/mL), were upregulated after CFA injection. However, 8-OaS treatment reversed these results, as our data for IL-1β (4.826 ± 0.121, 3.820 ± 0.203, 3.101 ± 0.214 pg/mL), IL-6 (9.573 ± 0.221, 7.516 ± 0.279, 7.426 ± 0.223 pg/mL), and TNF-a (30.833 ± 1.734, 16.611 ± 1.732, 15.278 ± 1.904 pg/mL) show (Fig. 4c, d). These data suggest that 8-OaS plays an effective role in anti-inflammation, which may be one of the ways in which it reduces chronic inflammatory pain.

### Effect of 8-OaS on Inflammation Signaling Pathways in Different Anxiety Models

The inflammation signaling pathway was activated upon CFA stimulation and acute stress, and we further studied which cytokines were responsible for this process. Western blotting was used to determine the expression levels of p-JNK, p-P38, p65, and TNF-α in the BLA. In the FSS model, the expression levels of p-JNK, p-P38 (Fig. 5a), p65, and TNF-α (Fig. 5b) were significantly enhanced in the BLA of mice after acute stress, compared with those in the control group. 8-OaS treatment resulted in the upregulation of p-JNK, p-P38 (Fig. 5a), p65, and TNF-α (Fig. 5b) expression.

**Figure:**
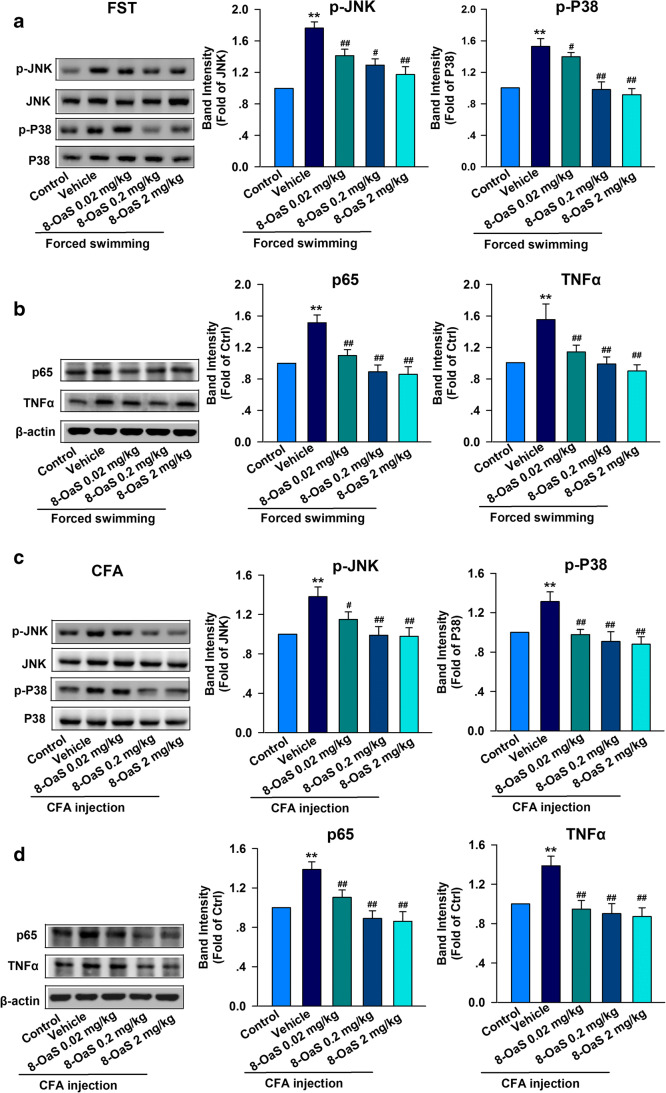

In the CFA model, compared with the control group, hind paw CFA injection resulted in the significant induction of p-JNK, p-P38 (Fig. 5c), p65, and TNF-α (Fig. 5d) proteins in the BLA, while 8-OaS administration for 2 weeks reduced the levels of p-JNK, p-P38 (Fig. 5c), p65, and TNF-α (Fig. 5d) relative to the control group. This suggests that 8-OaS is effective against inflammation, which here resulted in the alleviation of different anxiety models, including a CFA-induced chronic inflammatory pain model and an FSS-induced acute pain model.

## Discussion

In this study, we show that 8-OaS exhibits anxiolytic effects by using acute stress–induced and chronic inflammatory pain–induced models. Glutamatergic/GABAergic synaptic dysfunction caused by forced swimming was eliminated by 8-OaS. In addition, pro-inflammatory cytokine release and microglia activation were significantly attenuated by 8-OaS administration after CFA injection. Finally, p-JNK and p-P38 were involved in both the acute stress–induced and the chronic inflammatory pain–induced model. This suggests that the anxiolytic effect of 8-OaS was likely mediated by inhibiting the JNK/P38 MAPK pathway.

The *L*. *rotata* aqueous extract for oral administration was approved by the Food and Drug Administration of China (Beijing) in 1989 for pain therapy, and 8-OaS was considered to be the main effective ingredient, partially blocking formalin-induced tonic hyperalgesia as well as peripheral nerve injury–induced and bone cancer–induced mechanical allodynia (Zhu et al. 2014). Recently, it has been further demonstrated that 8-OaS alleviates neuropathic pain by inhibiting inflammatory responses in the spinal dorsal horn (Zhang et al. 2018). Numerous studies have provided evidence that the impact of a variety of inflammatory stimuli is related to mood and anxiety-related disorders. Clinical research also revealed that biomarkers of inflammation, such as inflammatory cytokines and acute-phase proteins, are reliably increased in a proportion of patients with major depressive disorder (MDD), bipolar disorder, anxiety disorders, and posttraumatic stress disorder (PTSD) (Felger 2018). Similar to that in analgesic experiments, we found that 8-OaS alleviated CFA-induced chronic inflammatory pain accompanying anxiety-like behaviors in mice. The activation of glial cells leads to the occurrence of neuroinflammation, which is mainly due to the early activation of microglia and the long-term activation of astrocytes. In addition, microglial injury may lead to depression, and drugs that inhibit microglial activation, such as minocycline and tumor necrosis factor alpha (TNF-α) inhibitors, are also considered effective antidepressants (Miller and Raison 2015; Yirmiya et al. 2015). In addition, microglia release and respond to several cytokines, including IL-1, IL-6, TNF-a, and IFN-c, which contribute to the maintenance of persistent pain states in autoimmune inflammation of the nervous system (Lee 2020; Santos et al. 2019). In this study, we observed that the upregulation of Iba-1 expression and the release of pro-inflammatory cytokines (IL-1β, IL-6, and TNF-α) in the BLA after CFA injection were decreased by 8-OaS, suggesting that the anxiolytic actions of 8-OaS are linked to the inactivation of microglia.

Anxiety is a general neurobehavioral correlate of various stressors, and both acute and chronic stress exposure could precipitate anxiety disorders (Chrousos 2009). In our study, forced swimming was used as acute stress to induce anxiety-like behaviors, which were reflected in behavioral changes in the OFT and EPM. Such stress responses can influence the immune system via microglial elimination/repopulation (Ray et al. 2017). Additionally, complex neurotransmitter networks provide a possible link between anxiogenesis and immunomodulation during stress. Our group previously demonstrated that glutamatergic and GABAergic systems were disrupted in a stress-induced model (Tian et al. 2013), where we used HPLC to examine glutamate and GABA concentrations and found increased glutamate and decreased GABA content in the BLA of FSS-treated mice, which was however reversed by 2 mg/kg 8-OaS. The excitatory and inhibitory (E/I) transmission balance is crucial for normal functioning of the brain, and enhanced excitatory or reduced inhibitory transmission can result in hyperexcitation and promote anxiety-like behaviors (Wu et al. 2008). It has also been reported that tonic GABAergic reduction is induced by social isolation stress (Matsumoto et al. 2007; Tian et al. 2013). Additionally, NMDA receptors, which are critical excitatory postsynaptic receptors, consist of three subunits: GluN1, GluN2, and GluN3 (Chen et al. 2019; Jeyifous et al. 2016). GluN2A and GluN2B are the most common NMDAR subtypes and play a significant role in the mammalian CNS, showing enhanced activity attributable to neurotransmitter hyperexcitability, a condition that is related to increased anxiety (Prager et al. 2014). PSD95 is a postsynaptic anchor protein that binds to NMDA and AMPA receptors (Jeyifous et al. 2016). Among the three subtypes of GABA receptors (GABA_A_, GABA_B_, and GABA_C_ subtypes), GABA_A_ receptors are typical ligand-gated ion channels that play the most important role in GABAergic inhibitory function, whereas subunits of GABA_A_ receptors—GABA_A_α2 and GABA_A_γ2—mediate anxiety in the BLA (Jeyifous et al. 2016; Möhler 2012). Consistent with these earlier findings, we found that the levels of the crucial postsynaptic proteins GluN2A, GluN2B, and PSD95 were enhanced, whereas the expression of GABA_A_-α2 and GABA_A_-γ2 receptors decreased in the BLA of mice with stress-induced anxiety. Furthermore, the increase in these receptors was reversed in the BLA of stressed animals after administration of 2 mg/kg 8-OaS. After FSS exposure, both neurotransmitters and postsynaptic receptors showed changes in excitation/inhibition signaling. This shows that, under acute stress, 8-OaS can improve anxiety-like behaviors by modifying the excitation/inhibition balance between neuronal signals.

TNF-α is a proinflammatory cytokine and a well-characterized indicator of neuroimmune inflammation. Besides, TNF-α regulates the trafficking of the AMPA receptor (AMPAR) on the synapse, by driving the rapid exocytosis on hippocampal pyramidal cells (Beattie et al. 2002; Ogoshi et al. 2005) and the endocytosis on the GABAergic MSNs of the striatum (Lewitus et al. 2014). Subsequent work has revealed that TNF-α can drive the simultaneous endocytosis of GABA-A receptors (GABARs), leading to a substantial shift in the excitation-to-inhibition (E/I) balance (Stellwagen et al. 2005). In our two anxiety models, TNF-α expression in the BLA was increased in both the forced swimming and the CFA injection condition. This suggests that there is a similar anxiety mechanism underlying the imbalance between neuroinflammation and synaptic transmission (Renna et al. 2018; ML et al. 2016; Ren and Dubner 2008; Kim et al. 2019; Zheng et al. 2011). Other studies have reported that *L*. *rotata can* promote microglia to secrete β-endorphin, with p38 MAPK signaling as a key linkage (Fan et al. 2016; Zhu et al. 2014). Here, we also found that the phosphorylation levels of p38 and JNK, part of the mitogen-activated protein kinase (MAPK) family, were markedly enhanced after chronic inflammation pain or acute stress. Fittingly, 8-OaS inhibited this signaling pathway in both anxiety models, resulting in the decreased expression of NF-κB p65 and TNF-α. Therefore, 8-OaS exhibited anxiolytic effects by inhibiting the JNK/P38 MAPK pathway, reducing the inflammatory response mediated by microglia activation, and altering synaptic function due to changes in neurotransmitter levels. However, the relationship between these processes is not clear, and further study is needed for clarification.

In conclusion, our findings demonstrate that 8-OaS alleviates anxiety-like behaviors in animal models of acute and chronic anxiety by regulating MAPK signaling pathways involved in anti-inflammatory activities and restoring the E/I balance. This study describes a new pharmacological effect of 8-OaS, thereby providing a theoretical basis for further clinical research on anxiolytic. However, other pathways may be involved in the anxiolytic activities of 8-OaS, and further experiments are needed to verify and expand the current findings.
